# Erythropoietin, transfusions, and outcomes of retinopathy of prematurity and brain injury in extremely preterm infants: A post hoc analysis of the Preterm Erythropoietin Neuroprotection Trial (PENUT)

**DOI:** 10.1371/journal.pone.0348061

**Published:** 2026-06-25

**Authors:** Nancy M. Fahim, Scott Lunos, Raghavendra B. Rao, Michael K. Georgieff, Sandra Juul, Ellen C. Ingolfsland

**Affiliations:** 1 Department of Pediatrics, Division of Neonatology, University of Minnesota, Minneapolis, Minnesota, United States of America; 2 Biostatistical Design and Analysis Center, Clinical and Translational Science Institute, University of Minnesota, Minneapolis, Minnesota, United States of America; 3 Department of Pediatrics, Division of Neonatology, University of Washington, Seattle, Washington, United States of America; Post Graduate Institute of Medical Education and Research, INDIA

## Abstract

**Background:**

Erythropoietin is perceived as both a neuroprotectant and a biomarker for hypoxic stress.

**Objective:**

To explore correlations between serum erythropoietin (Epo) concentrations, perinatal risk factors, red blood cell transfusions and recombinant human erythropoietin (rHuEpo) with outcomes including retinopathy of prematurity (ROP) and brain injury on magnetic resonance imaging (MRI) in extremely preterm infants.

**Methods:**

This is a post hoc analysis of data from the Preterm Erythropoietin Neuroprotection Trial of preterm infants born between 24 0/7 and 27 6/7 weeks gestation and randomized to placebo or rHuEpo treatment (N = 941). Serum Epo concentrations were collected within 24 hours (baseline) and at 7, 9, and 14 days. MRI was obtained at 36 weeks postmenstrual age (N = 220).

**Results:**

Baseline Epo concentrations negatively correlated with gestational age, delayed cord clamping, and Apgar scores, and positively correlated with intraventricular hemorrhage and risk of death. Neither endogenous Epo at baseline nor trajectories from birth to 14 days were associated with ROP. In the placebo group, Epo at 1 week of life (r = 0.26, p = 0.033) and 2-week area under the curve (r = 0.28, p = 0.019) positively correlated with white matter injury. In the treatment group, Epo at 14 days negatively correlated with white matter injury (r = −0.35, p = 0.004). Grey matter injury negatively correlated with baseline Epo in the placebo group (r = −0.27, p = 0.01) but positively correlated in the treatment group (r = 0.23, p = 0.047). Transfusions were associated with severe ROP (p < 0.0001) and total brain injury on MRI (p = 0.007). Transfusion volumes in the first week of life were associated with a greater risk of severe ROP in males (p = 0.0006).

**Conclusions:**

Endogenous Epo concentrations in preterm infants are influenced by perinatal variables and correlate with poor outcomes. The association of Epo with MRI results differed between placebo and rHuEpo treatment groups. Transfusions were associated with increased ROP and brain injury on MRI.

## Introduction

Endogenous serum erythropoietin (Epo) concentrations vary widely in preterm infants. Epo production is stimulated by hypoxia, mediated via hypoxia-inducible factor HIF-1α, and so may provide information about the intrauterine environment, with higher Epo levels reflecting relative hypoxemia [1].

Early endogenous Epo concentrations during the first 2 weeks of life may convey information about risks of bowel, pulmonary, and retinal diseases in extremely preterm infants born before the 28^th^ week of gestation [2]. Early elevated endogenous Epo concentrations in the extremely preterm infant have also been associated with inflammation-related proteins [3], a higher risk of lower Mental and/or Psychomotor Development Indices and microcephaly at 2 years [4], and cognitive impairment at 10 years [5]. Elevated Epo at baseline predicted increased risk of death or severe disability at 22–26 months corrected age (CA) in a large cohort of infants born extremely premature in the Preterm Erythropoietin Neuroprotection Trial (PENUT) [6].

Epo is also an erythropoietic stimulating agent and has been used in the extremely premature infant to decrease packed red blood cell (pRBC) transfusions [7,8]. This is of particular significance as recent yet conflicting literature is emerging regarding potential adverse events associated with pRBC transfusions in that population [9–12].

In a small cohort study (n = 27) of preterm infants, we recently showed that higher endogenous Epo concentrations in the first 2 weeks of life were associated with lower Apgar scores at birth and increased severity of retinopathy of prematurity (ROP) and brain injury assessed using MRI at 34–40 weeks postmenstrual age (PMA) [13]. In addition, the number of pRBC transfusions correlated with increased white matter injury and stage of ROP [13].

The objective of this study was to investigate endogenous Epo concentrations during the first two weeks of life in a large cohort (n = 941) of extremely preterm infants who were enrolled in the PENUT Trial [14]. We aimed to determine potential associations between Epo concentrations, perinatal risk factors, interventions including pRBC transfusions and recombinant human erythropoietin (rHuEpo) therapy, and outcomes including ROP and brain injury using MRI near term corrected gestation. We hypothesized that increased endogenous Epo concentrations in the first 2 weeks of life are associated with complications of pregnancy, the risk and severity of ROP, and increased brain injury on imaging. We further hypothesized that pRBC transfusions during NICU stay would be associated with increased adverse outcomes of ROP and brain injury on MRI.

## Materials and methods

The PENUT trial (NCT01378273, PI Sandra Juul) was approved by the institutional review boards at all participating sites: All Childrens Hospital, St. Petersburg, FL (Johns Hopkins Office of Human Subjects Research Institutional Review Boards IRB00060341); Beth Israel Deaconess Medical Center, Boston, MA (Boston Children’s Office of Clinical Investigation IRBP00008415); Childrens Hospitals and Clinics of Minnesota, Minneapolis, MN (Children’s Hospitals and Clinicals of Minnesota IRB# 1308−079); Childrens Hospitals and Clinics of Minnesota, St. Paul, MN (Children’s Hospitals and Clinicals of Minnesota IRB# 1308−079); Florida Hospital Orlando, Orlando, FL (Florida Hospital Office of Research Administration IRB-6055); Johns Hopkins University, Baltimore, MD (Johns Hopkins Office of Human Subjects Research Institutional Review Boards IRB00026419); Methodist Childrens Hospital, San Antonio, TX (Methodist Healthcare IRB 440455−3); Maria Fareri Childrens Hospital, Westchester, NY (New York Medical College Office of Research Administration IRB# 10265); South Miami Hospital, Miami, FL (Baptist Health South Florida IRB# 15−037); Prentice Womens Hospital of Northwestern Memorial Hospital, Chicago, IL (Ann and Robert H Lurie Children’s Hospital of Chicago 2013−1543); University of Florida, Gainsville, FL (University of Florida #142−2013); Childrens Hospital of the University of Illinois (University of Illinois Chicago Office for the Protection of Research Subjects #2013−0835); University of Minnesota Childrens Hospital, Minneapolis, MN (University of Minnesota 1308M41201); University of New Mexico, Albuquerque, NM (University of New Mexico Health Sciences Center Human Research Protections Office #13−464, Presbyterian Healthcare Services Institutional Review Board 662482−1); University of Arkansas, Fayetteville, AR (University of Arkansas for Medical Sciences IRB #202449); Kosair Childrens Hospital, Louisville, KY (Norton Healthcare Office of Research Administration NHORA#13-N0095); University of Utah, Salt Lake City, UT (University of Utah IRB #00063749, Intermountain Healthcare IRB #1024761); University of Washington, Seattle, WA (University of Washington Human Subjects Division STUDY00007467); Wake Forest University, Wake Forest, NC (Wake Forest University Health Sciences IRB #00023079).

All participants’ parents in the PENUT study at all sites provided written informed consent.

### Enrollment

**Fig 1:**
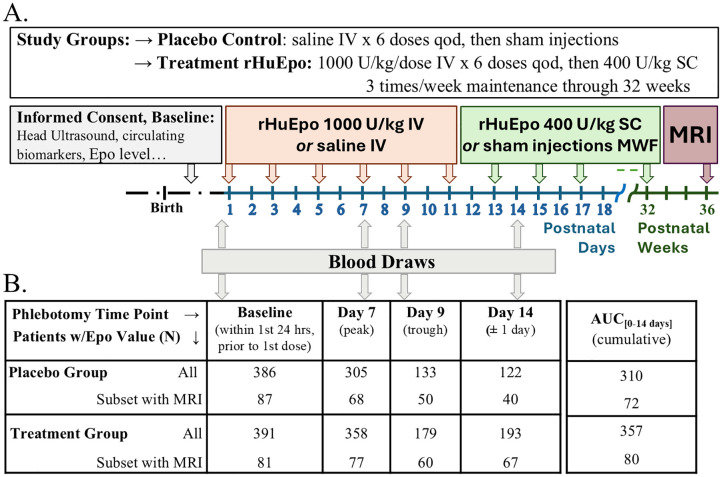
PENUT trial treatment overview and phlebotomy timing, with sample size for post hoc epo and MRI analyses. (A) Study overview of treatment dosing algorithm for both groups, with timing of MRI and blood draws for Epo concentration assessment. (B) Sample sizes available for these post hoc analyses. Abbreviations: qod: every other day, IV: intravenous, SC: subcutaneous, MWF: Monday, Wednesday, Friday, U/kg: unit/ kilogram. Caption Credit: (A) Based on [14] Supplementary Material, Protocol, with major revisions and additions (https://www-nejm-org.ezp3.lib.umn.edu/doi/suppl/10.1056/NEJMoa1907423/suppl_file/nejmoa1907423_protocol.pdf).

The PENUT Trial was a multicenter, randomized, double-blinded trial of high-dose rHuEpo for preterm neuroprotection, enrolling 941 infants born between 24 0/7 and 27 6/7 weeks gestation after written parental informed consent at 19 sites throughout the United States [14]. Enrollment occurred from December 2013 through September 2016. The primary outcome was death or severe neurodevelopmental impairment (NDI), defined as severe cerebral palsy or a composite motor or cognitive score of <70 on the Bayley Scales of infant and toddler development, 3rd edition (BSID-III), at 22–26 months corrected age (CA). Study participants were randomized to receive either rHuEpo 1000 U/Kg or placebo (saline), with the initial dose given within the first 24 hours after birth, and then every 48 hours for a total of six doses throughout the first 2 weeks after birth. Thereafter, infants received maintenance subcutaneous injections of rHuEpo at a dose 400 U/kg or sham injections, 3 times per week through 32 weeks and 6 days post menstrual age (PMA); 477 infants were assigned to the rHuEpo group and 464 to the placebo group. Site-specific transfusion practices were allowed. Details including enrollment, study design and oversight, randomization, iron supplementation, primary and secondary outcomes, adverse events, etc. are published in Juul et al, 2020 [14]. The initial treatment algorithm and timing of serum draws and MRI assessment through the first 36 weeks are illustrated in Fig 1A.

### Data collection

De-identified human data from the PENUT Trial [14] was obtained from the NINDS Data Archive (https://www.ninds.nih.gov) on September 28, 2021. The authors had no access to information that could identify individual participants during or after data collection. The study dataset included the modified intent-to-treat (mITT) population of rHuEpo-treated and placebo-treated control infants who were appropriately consented, underwent randomization, and received the first dose of rHuEpo or placebo. Data collected included pregnancy, delivery, maternal and infant characteristics, and infant outcomes.

Serum Epo concentrations were collected within the first 24 hours (baseline) and at 7 (peak), 9 (trough), and 14 days after birth per protocol. Plasma-separated samples were stored at -70°C and shipped on dry ice to the University of Washington for quantification using Meso Scale Discovery electro-chemiluminescence-based assays (Human Epo Base Kit or U-Plex, Meso Scale Discovery, Rockville, MD).

Baseline Epo concentrations were collected prior to the first dose of rHuEpo or placebo and therefore reflect endogenous Epo concentrations in both groups. Subsequent Epo concentrations over time continue to reflect endogenous Epo in the placebo group, but not in the treatment group. After the first dose, serum Epo concentrations in the treatment group instead reflect a combination of high dose exogenous and (possibly suppressed) endogenous Epo (see Fig 1A), which complicates interpretation of treatment group Epo values over time. In this ad hoc analysis, subjects missing more than 2 of the 4 serum Epo samples required per-protocol were excluded from some analyses.

ROP was screened and treated according to the 2006 American Academy of Pediatrics practice guideline [15], which is based on findings of the Early Treatment of Retinopathy study [16]. ROP severity was reported according to the International Classification of ROP (ICROP) [17]. ROP outcomes of any ROP or severe ROP, defined as ROP requiring laser or bevacizumab therapy, were used in the PENUT study and in this analysis.

MRI brain imaging at 36 0/7–36 6/7 weeks PMA was performed on a subset of subjects (n=220 scans meeting imaging standards, n=110 in each group) at eight study sites identified at trial initiation. Acquisition protocols were standardized across sites. T1 and T2 weighted and gradient images were blindly scored by 2 neuroradiologists independently utilizing the scoring system of Kidokoro et al [18]. In this analysis, only the subset of subjects with MRI data were included for the brain injury imaging endpoints.

### Outcome variables

Epo levels were measured at specific time points and log-transformed for analysis. We analyzed the association between Epo concentrations at each time point and the area under the curve (a cumulative measure of concentration over time) during the first 2 weeks of life (AUC_[Epo] 0–2weeks_) and various maternal and infant characteristics, delivery, and infant outcomes. These include: Apgar score, delayed cord clamping, initial hemoglobin, pRBC transfusions, necrotizing enterocolitis (NEC), ROP presence and severity, intraventricular hemorrhage (IVH) on day 7–9 ultrasound (US), and death. We also looked at the relationship between Epo levels and outcomes of brain injury score by MRI at 36 weeks PMA and developmental milestones assessed by the Bayley Scales (BSIDIII) at 22–26 months corrected age.

### Statistical analysis

Descriptive statistics (means, standard deviations, medians, ranges, counts and percents) were used to summarize the data. Generalized estimating equations (GEE) models for continuous (linear) or binary (logistic) outcomes with robust standard errors were used to analyze associations between characteristics/outcomes and Epo values (at each time point and AUC), adjusted for gestational age at birth and enrollment site (and treatment group where applicable). GEE takes into account potential correlation within siblings of the same pregnancy. GEE models were also used to compare pRBC transfusion exposure between treatment groups, adjusted for gestational age and site, and associations between pRBC transfusion and outcomes including ROP and brain injury scores, adjusted for gestational age, site, sex and treatment group.

For binary outcome models, odds ratios (95% confidence intervals) and p-values are reported. For continuous outcome models, estimates, SE and p-values are reported. We also calculated Spearman correlation coefficients between Epo measures and continuous characteristics and outcomes and adjusted for gestational age (or hematocrit where noted). Interaction tests were performed using GEE and linear regression models and were adjusted for sex, gestational age, and enrollment site where noted. Two group t-tests were used to compare Epo concentrations between transfusion status (any vs none) at each time point. Two-tailed p-values less than 0.05 were considered statistically significant and were not adjusted for multiple comparisons since the analyses are exploratory. All available data were used for the analysis and missing data were not imputed. SAS V9.4 (SAS Institute Inc., Cary, NC) was used for analysis.

## Results

### Study population demographics and data collection

Baseline maternal, pregnancy/delivery, and infant characteristics of patients enrolled in the PENUT trial (Epo group n = 476, and placebo group n = 460) are provided in the S1 Appendix, adapted from Juul et al [14]. Characteristics of the subset with MRI are reported by Mayock et al [19]. The evaluable sample size in each group with Epo values at each time point and the subset with MRI are shown in Fig 1B.

### Association of Epo with perinatal variables

Baseline Epo concentrations were drawn at a median age of 17 hrs after birth (11–22 hrs interquartile range) and were available from 777 infants (Epo group n = 391, and placebo group n = 386). No associations were observed between Epo concentrations and characteristics of pregnancy (maternal age, race, multiple fetuses, preeclampsia) or delivery (S2 Appendix), although there was a trend (p = 0.0504 for both groups combined) towards higher baseline Epo in pregnancies with multiple fetuses and a trend (p = 0.0502 in the placebo group) towards higher endogenous Epo AUC_[0-14d]_ with pregnancy induced hypertension (S2 Appendix).

Baseline Epo concentrations were negatively associated with gestational age for all patients (p = 0.0006 by GEE linear models, Table 1; r = −0.14, p < 0.0001 by correlation, S3 Appendix), a relationship that persisted after adjusting for hematocrit (p = 0.002, Table 1). Epo concentrations at one-week were negatively associated with birth weight in the placebo group (p = 0.0002 by GEE linear models, Table 1, and r = −0.15, p = 0.010 by correlation, Table 2).

**Table 1:** Association of endogenous epo concentration with infant characteristics and clinical outcomes in all patients at baseline and in the placebo group over time (GEE).

	Baseline Epo (Both groups)	Day 7 Epo (Placebo group)	Day 9 Epo (Placebo group)	Day 14 Epo (Placebo group)	Epo AUC_[0-14d]_ (Placebo group)
**Infant characteristics** Estimate (SE)					
Sex – female	−0.08 (0.13) p = 0.5269	**0.28 (0.12)** **p = 0.0210**	0.26 (0.20) p = 0.1969	0.05 (0.18) p = 0.7632	0.08 (0.13) p = 0.5264
Gestational age (wks)	**−0.19 (0.06)** **p = 0.0006**	0.07 (0.05) p = 0.2117	−0.09 (0.08) p = 0.2473	−0.02 (0.10) p = 0.8136	−0.09 (0.06) p = 0.1442
Gestational age (wks) (adjusted for hematocrit)	**−0.18 (0.06)** **p = 0.0022**	0.07 (0.05) p = 0.2129	−0.09 (0.08) p = 0.2729	−0.04 (0.10) p = 0.6730	−0.07 (0.06) p = 0.2618
Birth weight (g)	0.00 (0.00) p = 0.1487	**−0.002 (0.0004)** **p = 0.0002**	−0.001 (0.001) p = 0.1679	−0.00 (0.001) p = 0.9938	−0.001 (0.00) p = 0.0918
Birth weight <10^th^ percentile for gestational age	−0.09 (0.14) p = 0.5253	0.25 (0.16) p = 0.1380	0.11 (0.25) p = 0.6540	−0.03 (0.19) p = 0.8544	0.04 (0.17) p = 0.8295
Apgar score at 1 min	−0.05 (0.03) p = 0.0703	−0.02 (0.03) p = 0.5304	−0.04 (0.05) p = 0.4468	−0.05 (0.05) p = 0.2401	**−0.09 (0.03)** **p = 0.0080**
Apgar score at 5 min	−0.05 (0.03) p = 0.1263	−0.01 (0.03) p = 0.7051	−0.05 (0.05) p = 0.4045	−0.08 (0.05) p = 0.0866	−0.06 (0.04) p = 0.0857
Apgar score <5 at 5 min	0.22 (0.15) p = 0.1426	−0.06 (0.15) p = 0.6721	0.11 (0.27) p = 0.6811	0.35 (0.26) p = 0.1933	0.19 (0.20) p = 0.3383
Delayed cord clamping >30 Seconds	**−0.37 (0.16)** **p = 0.0249**	−0.02 (0.16) p = 0.9196	0.20 (0.24) p = 0.4139	**−0.60 (0.22)** **p = 0.0109**	−0.14 (0.19) p = 0.4634
Delayed cord clamping (adjusted for hematocrit)	**−0.35 (0.17)** **p = 0.0421**	−0.01 (0.16) p = 0.9367	0.22 (0.24) p = 0.3670	**−0.63 (0.20)** **p = 0.0057**	−0.09 (0.20) p = 0.6450
**Infant Outcome** OR (95% CI)					
Death	**1.16 (1.03-1.31)** **p = 0.0206**	1.00 (0.64-1.53) p = 0.9896	0.97 (0.48-1.98) p = 0.9437	NA	1.13 (0.79-1.61) p = 0.5002
ROP					
Severe (grade ≥ 3)	1.13 (0.99-1.28) p = 0.0714	1.01 (0.76-1.35) p = 0.9203	1.14 (0.75-1.71) p = 0.5438	1.09 (0.62-1.92) p = 0.7723	1.20 (0.95-1.51) p = 0.1352
Any, all grades	1.04 (0.94-1.14) p = 0.4961	1.04 (0.80-1.34) p = 0.7777	1.21 (0.91-1.61) p = 0.2053	0.81 (0.56-1.16) p = 0.2491	1.01 (0.81-1.26) p = 0.9268
NEC					
Grade 2b or 3 (Bell’s stage)	1.07 (0.92-1.25) p = 0.9199	0.92 (0.59-1.41) p = 0.6736	0.84 (0.38-1.86) p = 0.6838	1.14 (0.27-4.87) p = 0.8606	1.10 (0.83-1.46) p = 0.5230
Any, all grades	0.99 (0.87-1.14) p = 0.9396	1.02 (0.75-1.37) p = 0.9199	1.22 (0.76-1.98) p = 0.3906	1.65 (0.81-3.38) p = 0.1868	1.09 (0.87-1.37) p = 0.4741
Intraventricular hemorrhage					
Grade III or IV	**1.32 (1.14–1.54) p = 0.0009**	0.76 (0.53-1.08) p = 0.1198	1.33 (0.75-2.35) p = 0.3437	0.98 (0.49-1.94) p = 0.9432	1.25 (0.92-1.72) p = 0.1859
Any, all grades	**1.21 (1.10-1.34)** **p < 0.0001**	1.07 (0.82-1.39) p = 0.6088	**1.54 (1.04-2.26)** **p = 0.0200**	1.31 (0.82-2.11) p = 0.2069	1.17 (0.95-1.45) p = 0.1194
Severe developmental impairment					
BSID-III composite cognitive score < 70	1.10 (0.83-1.44) p = 0.5651	0.84 (0.54-1.30) p = 0.3966	0.80 (0.47-1.36) p = 0.4459	1.56 (0.63-3.87) p = 0.4176	1.15 (0.81-1.63) p = 0.4569
BSID-III composite motor score <70	1.13 (0.92-1.39) p = 0.3213	1.01 (0.73-1.38) p = 0.9640	0.99 (0.67-1.48) p = 0.9764	1.55 (0.55-4.34) p = 0.4796	1.19 (0.93-1.52) p = 0.1792
BSID-III composite language score < 70	1.09 (0.93-1.29) p = 0.3356	0.81 (0.54-1.21) p = 0.2628	0.77 (0.48-1.24) p = 0.3409	0.88 (0.40-1.93) p = 0.7522	1.13 (0.85-1.49) p = 0.4158
Moderate-to-severe developmental impairment					
BSID-III composite cognitive <85	1.06 (0.94-1.20) p = 0.3810	0.76 (0.53-1.09) p = 0.1136	0.73 (0.53-1.01) p = 0.0665	0.74 (0.47-1.18) p = 0.1990	1.05 (0.85-1.30) p = 0.6302
BSID-III composite motor score <85	1.07 (0.95-1.21) p = 0.2522	0.77 (0.52-1.12) p = 0.1265	0.93 (0.68-1.27) p = 0.6452	1.55 (0.55-4.34) p = 0.4796	0.83 (0.53-1.29) p = 0.3879
BSID-III composite language score < 85	1.05 (0.94-1.16) p = 0.4040	0.92 (0.72-1.17) p = 0.4974	0.80 (0.59-1.09) p = 0.1658	0.77 (0.51-1.17) p = 0.1898	1.08 (0.88-1.32) p = 0.4472
BSID-III composite score					
Cognitive, Estimate (SE)	−0.49 (0.49) p = 0.3290	0.38 (0.81) p = 0.6479	0.94 (1.06) p = 0.3824	0.99 (1.77) p = 0.5805	−0.73 (0.74) p = 0.3135
Motor, Estimate (SE)	−0.47 (0.52) p = 0.3688	0.31 (0.87) p = 0.7225	0.00 (1.25) p = 0.9982	−0.42 (2.00) p = 0.8326	−0.76 (0.83) p = 0.3434
Language, Estimate (SE)	−0.46 (0.52) p = 0.3804	0.61 (0.99) p = 0.5559	0.68 (1.34) p = 0.6213	1.12 (2.29) p = 0.6283	−0.90 (0.85) p = 0.2719
Cerebral palsy, score 1–5 (Yes vs No), OR (95% CI)	1.01 (0.87-1.18) p = 0.8894	0.78 (0.54-1.13) p = 0.1721	1.24 (0.76-2.01) p = 0.3835	0.97 (0.34-2.72) p = 0.9471	1.15 (0.90-1.46) p = 0.2774

Estimates and p-values are from GEE models using the natural logarithm of Epo concentration at each time point. These models account for potential correlation within siblings and are adjusted for gestational age at birth and site. For the analysis of Baseline Epo using the combined groups, treatment group was also included as a fixed effect. For binary outcome models, odds ratios (95% confidence intervals) and p-values are reported. For continuous outcome models, estimates, SE and p-values are reported. These estimates represent average change in outcome based on a one-unit change in ln(Epo). Intraventricular hemorrhage (IVH) was determined at day 7–9 by ultrasound. Abbreviations: OR odds ratio, SE standard error, NEC necrotizing enterocolitis, ROP retinopathy of prematurity, BSID-III Bayley Scales of infant and toddler development, 3rd edition, assessed at 22–26 months CA.

**Table 2:** Spearman partial correlation of endogenous epo concentrations with infant characteristics and outcomes in all patients at baseline and in the placebo group over time, adjusted for gestational age (GA).

	Baseline Epo (Both groups)	Baseline Epo (Placebo group)	Day 7 Epo (Placebo group)	Day 9 Epo (Placebo group)	Day 14 Epo (Placebo group)	Epo AUC_[0-14d]_ (Placebo group)
Birth Weight	**r = 0.09** **p = 0.0170** n = 777	r = 0.06 p = 0.2478 n = 386	**r = −0.15** **p = 0.0102** n = 305	r = −0.11 p = 0.2251 n = 133	r = 0.01 p = 0.9322 n = 122	r = −0.04 p = 0.4461 n = 310
First Hematocrit	**r = −0.09** **p = 0.0090** n = 775	**r = −0.21** **p < 0.0001** n = 385	r = 0.02 p = 0.6977 n = 304	r = −0.09 p = 0.3079 n = 132	r = 0.10 p = 0.2703 n = 121	r = −0.09 p = 0.1082 n = 309
Apgar at 1 minute	**r = −0.10** **p = 0.0054** n = 774	**r = −0.18** **p = 0.0004** n = 385	r = 0.04 p = 0.4520 n = 305	r = −0.09 p = 0.2870 n = 133	r = −0.13 p = 0.1495 n = 122	**r = −0.14** **p = 0.0160** n = 310
Apgar at 5 minutes	r = −0.06 p = 0.0884 n = 775	r = −0.08 p = 0.1067 n = 386	r = 0.01 p = 0.8517 n = 305	r = −0.09 p = 0.2979 n = 133	r = −0.16 p = 0.0890 n = 122	**r = −0.12** **p = 0.0385** n = 310
ROP Stage (max of both eyes)	r = 0.03 p = 0.4862 n = 700	r = 0.00 p = 0.9353 n = 353	r = −0.01 p = 0.8935 n = 295	r = 0.09 p = 0.3291 n = 130	r = −0.09 p = 0.3297 n = 120	r = 0.03 p = 0.6122 n = 300
MRI Total Injury Score	r = 0.03 p = 0.6608 n = 168	r = −0.03 p = 0.7533 n = 87	r = 0.16 p = 0.1965 n = 68	r = 0.15 p = 0.3009 n = 50	r = 0.10 p = 0.5372 n = 40	r = 0.06 p = 0.6115 n = 72
MRI White Matter Score	r = 0.02 p = 0.7693 n = 168	r = 0.03 p = 0.7742 n = 87	**r = 0.26** **p = 0.0331** n = 68	r = 0.09 p = 0.5287 n = 50	r = 0.07 p = 0.6551 n = 40	**r = 0.28** **p = 0.0191** n = 72
MRI Grey Matter Score	r = 0.00 p = 0.9572 n = 166	**r = −0.27** **p = 0.0106** n = 87	r = −0.19 p = 0.1169 n = 68	r = 0.11 p = 0.4371 n = 50	r = −0.06 p = 0.7126 n = 40	r = −0.18 p = 0.1292 n = 72

Spearman partial correlation coefficient estimate and p-value are presented for association of ln(Epo) at birth for all patients and in the placebo group at each time point and AUC with perinatal variables and ROP and MRI outcomes, adjusted for gestational age (GA). Analysis subset includes only subjects with Epo values at the respective time points (and at least 2 values for AUC). Abbreviations: AUC: area under the curve; ROP: retinopathy of prematurity.

There was a negative association between delayed cord clamping and Epo concentrations in all patients at baseline (p = 0.025) and in the placebo group at 14 days (p = 0.011; Table 1), and these associations persisted after adjusting for hematocrit (p = 0.042 and p = 0.006, respectively). Higher baseline Epo concentrations correlated with lower initial hematocrit levels in all patients (r = −0.09, p = 0.009; Table 2).

Baseline Epo concentrations negatively correlated with 1 minute Apgar score in all patients (r = −0.10, p = 0.005, Table 2). In the placebo group, the 2-week area under the curve of Epo concentrations (Epo AUC_[0-14d]_) negatively correlated with Apgar scores at both 1 minute (r = −0.14, p = 0.016) and 5 minutes (r = −0.12, p = 0.039; Table 2).

### Association of Epo with outcomes

No associations were observed between Epo concentrations and NEC or 2-year BSID-III composite scores in any group (Table 1). Higher baseline Epo concentrations in all patients were associated with increased risk of death (p = 0.021), with an adjusted odds ratio (OR) of 1.16 (1.03–1.31), Table 1.

#### Epo and retinopathy of prematurity.

The unadjusted correlation between baseline Epo concentrations and ROP outcomes differed between groups. Baseline Epo concentrations positively correlated with ROP stage in the treatment group (r = 0.12, p = 0.029, S4 Appendix) but not in the placebo group (r = 0.03, p = 0.551, S3 Appendix). However, this correlation did not persist after correcting for gestational age (Tables 2 and 3).

**Table 3:** Spearman partial correlation of epo concentration with outcomes in the treatment group over time, adjusted for gestational age (GA).

	Baseline Epo (treatment group)	Day 7 Epo (treatment group)	Day 9 Epo (treatment group)	Day 14 Epo (treatment group)	AUC_Epo[0-14d]_ (treatment group)
ROP Stage (max of both eyes)	r = 0.05 p = 0.3995 n = 347	r = −0.04 p = 0.4369 n = 331	r = −0.07 p = 0.3913 n = 170	r = 0.06 p = 0.4487 n = 187	r = −0.00 p = 0.9295 n = 331
MRI Total Injury Score	r = 0.11 p = 0.3192 n = 81	r = −0.06 p = 0.5893 n = 77	r = −0.16 p = 0.2125 n = 60	r = −0.24 p = 0.0539 n = 67	r = −0.03 p = 0.7909 n = 80
MRI White Matter Injury Score	r = 0.03 p = 0.7967 n = 81	r = 0.00 p = 0.9666 n = 77	r = −0.09 p = 0.4953 n = 60	**r = −0.35** **p = 0.0037** n = 67	r = 0.01 p = 0.9280 n = 80
MRI Grey Matter Injury Score	**r = 0.23** **p = 0.0474** n = 79	r = −0.13 p = 0.2567 n = 75	r = −0.21 p = 0.1069 n = 59	r = 0.23 p = 0.0619 n = 66	r = −0.06 p = 0.6251 n = 78

Spearman partial correlation coefficient estimate and p-value are presented at each time point for correlation of ln(Epo) with ROP and brain injury on MRI in the rHuEpo treatment group, adjusted for gestational age. Analysis subset includes only subjects with Epo values at the respective time points (and at least 2 values for AUC). Abbreviations: AUC: area under the curve, ROP: retinopathy of prematurity. Epo values after baseline and AUC reflect a combination of exogenous and endogenous Epo in the treatment group.

The results of interaction tests and ROP outcomes are reported in S5 Appendix. The interaction test to determine if there was an association between endogenous Epo concentrations at baseline and treatment with rHuEpo and ROP outcomes was negative (p > 0.5 for both severe ROP and any ROP; Table S5a in S5 Appendix). To further determine if the endogenous Epo trajectory affected ROP outcomes, interaction tests were performed testing endogenous Epo levels at birth and day 14 and ROP outcomes in the placebo group (Table S5b in S5 Appendix). This model was adjusted for hematocrit levels due to a prior report of association between endogenous Epo levels in the first week of life and anemia [20]. Additional t-tests examining Epo and binary outcomes of any or severe ROP were also negative, although trends were observed (0.05 < p < 0.06, S5 Tables S5c and S5d in S5 Appendix) in both groups combined that suggested a possible correlation between higher baseline Epo and increased risk of severe ROP (grade ≥ 3). However, the results of all interaction tests were not significant.

#### Epo and brain injury on imaging.

Higher baseline Epo concentrations were associated with an increased incidence of intraventricular hemorrhage (IVH) at day 7–9 in all patients (p < 0.0001) and with a higher incidence of grades III and IV hemorrhage (p = 0.0009; Table 1).

The association of Epo concentrations with MRI results differed between treatment and placebo groups:

*White Matter Injury:* Higher endogenous Epo concentrations at 1 week and the 2-week AUC (Epo AUC_[0-14d]_) correlated with increased white matter injury score in the placebo group (r = 0.26, p = 0.033 and r = 0.28, p = 0.019 respectively; Table 2). However, this correlation was negative in the treatment group, where higher Epo concentrations at 14 days (reflecting rHuEpo) were associated with decreased white matter injury (r = −0.35, p = 0.004; Table 3).

*Grey Matter Injury:* Higher baseline Epo concentrations negatively correlated with grey matter injury in the placebo group (r = −0.27, p = 0.011; Table 2), suggesting that higher endogenous Epo concentrations were associated with less grey matter injury. However, this correlation was positive in the treatment group, where higher baseline Epo concentrations correlated with increased grey matter injury score (r = 0.23, p = 0.047; Table 3). Results using GEE models are provided in S6 Appendix.

### Association of transfusions with epo concentrations and outcomes

#### Transfusions and Epo concentrations.

The mean Epo concentrations in pRBC-transfused and non-transfused subsets within each treatment group were similar (Table 4) and showed no statistically significant differences, albeit Epo concentrations were only measured until 14 days of life. Independent of transfusion status, the approximately 5–10 times higher Epo concentrations in the treatment group after baseline (compared to placebo) are from exogenous rHuEpo.

**Table 4:** Epo concentration by transfusion status within each Group.

	Placebo group			Treatment group		
Time Point	No Transfusion Mean (SD) n = 43	Any Transfusion Mean (SD) n = 417	T-test p-value	No Transfusion Mean (SD) n = 114	Any Transfusion Mean (SD) n = 362	T-test p-value
Baseline	1.8 (1.7)	2.2 (1.5)	0.2274	2.2 (1.9)	2.5 (1.9)	0.2087
Day 7	0.8 (0.8)	0.9 (1.1)	0.9141	8.0 (1.4)	7.8 (1.5)	0.3995
Day 9	1.1 (1.5)	1.4 (1.2)	0.3899	2.8 (1.3)	2.8 (1.3)	0.7931
Day 14	1.2 (0.9)	1.4 (1.1)	0.3870	3.3 (1.2)	3.2 (1.4)	0.7016
AUC_[0-14d]_	3.6 (1.4)	3.9 (1.3)	0.3460	9.4 (1.0)	9.2 (1.3)	0.0869

Ln (Epo) concentrations at each time point between 0 and 14 days and 2-week area under the curve (AUC_[0-14d]_) are presented as mean (SD). AUC calculated by trapezoidal rule. All available data from the modified ITT sample was used in these analyses. Not all patients had Epo values at each time point. Epo values after baseline and AUC reflect a combination of exogenous and endogenous Epo in the treatment group.

#### Transfusions and ROP.

Transfusions were associated with increased incidence and severity of ROP. Any transfusion received was associated with increased risk of developing any ROP (adjusted OR = 3.37 (2.16–5.27), p < 0.0001) and severe ROP (adjusted OR =12.09 (3.06–47.75), p < 0.0001, Table 5). The number of transfusions received (p < 0.0001), the overall pRBC volume (p ≤ 0.0001) and the volume received during the first week of life (p ≤ 0.0007) were all associated with severe ROP and with any ROP (Table 5). The association between transfusions and ROP did not differ by treatment group. When evaluated for sex differences (interaction p = 0.013), transfusion volumes in the first week of life were associated with severe ROP in males, with adjusted OR= 1.04 (1.02–1.06), p = 0.0006, but not females (adjusted OR=1.01 (0.99–1.04), p = 0.318). All other sex by transfusion interactions were not significant. The interaction tests to determine if there was an association between baseline endogenous Epo levels with number of transfusions and ROP outcomes were negative (S7 Appendix).

**Table 5:** Associations between pRBC Transfusions and ROP (all patients).

	Severe ROP OR (95% CI)	Any ROP OR (95% CI)
Any Transfusion	12.09 (3.06-47.75) **p < 0.0001**	3.37 (2.16-5.27) **p < 0.0001**
Number of Transfusions	1.16 (1.11-1.22) **p < 0.0001**	1.16 (1.09-1.23) **p < 0.0001**
Volume Overall	1.05 (1.03-1.08) **p < 0.0001**	1.04 (1.01-1.08) **p = 0.0001**
Volume first week	1.03 (1.02-1.05) **P = 0.0007**	1.04 (1.02-1.05) **p < 0.0001**

Adjusted odds ratio (OR) with confidence interval (CI) and p-value are from separate GEE models adjusted for treatment group, sex, GA and site. ORs are based on a 1-unit change, except Volume Overall, which is based on a 10-unit change. All available data from the mITT sample (n = 936) was used for these analyses.

#### Transfusions and brain injury on MRI.

The number and volume of pRBC transfusions per treatment arm throughout the NICU stay are shown in Table 6. In the placebo group, 91% of patients received at least one pRBC transfusion, while 76% received at least one pRBC transfusion in the treatment group (p < 0.0001), slightly more than reported by Juul et al [21], which included transfusions only through 12 weeks of age.

**Table 6:** pRBC transfusion exposure by treatment group during NICU stay.

	Placebo Group n = 460	Treatment Group n = 476	P-value
Any Transfusion, n (%) Yes No	417 (91) 43 (9)	362 (76) 114 (24)	**<0.0001**
Number of Transfusions, mean (SD)	5.8 (5.1)	3.9 (4.8)	**<0.0001**
Transfused Volume (mL), mean (SD)			
Total	86.8 (94.2)	56.8 (91.3)	**<0.0001**
DOL 0–6	14.0 (14.9)	13.2 (15.4)	0.8606
DOL 0–13	24.9 (21.3)	22.7 (27.9)	0.3961
DOL ≥ 7	72.7 (90.3)	43.5 (85.2)	**<0.0001**

P-values are from separate GEE models comparing treatment groups adjusted for GA and site. DOL: day of life.

There was an association between transfusions and MRI injury scores (Table 7). Any transfusion (≥1 vs. none) was associated with increased overall total injury score (p = 0.0067). Total brain injury and white matter injury scores were associated with the number of transfusions received (p < 0.0001, p = 0.0014, respectively), the overall pRBC volume (p < 0.0001, p = 0.0037), and the total volume received during the first week of life (p = 0.0009, p = 0.0031). The association between transfusions and MRI score did not differ by treatment group or by sex. Transfusion volumes in the first week of life were similar between treatment and placebo groups (14 vs 13 mL, p = 0.86, Table 6).

**Table 7:** Association between pRBC transfusion and brain injury by MRI in all patients (both groups combined).

	Total Injury Score	White Matter Score	Grey Matter Score
Any Transfusion	1.14 (0.40) **p = 0.0067**	0.40 (0.23) p = 0.0824	0.02 (0.05) p = 0.7663
Number of Transfusions	0.26 (0.05) **p < 0.0001**	0.10 (0.03) **p = 0.0014**	−0.00 (0.005) p = 0.6494
Volume Overall	0.01 (0.003) **p < 0.0001**	0.005 (0.002) **p = 0.0037**	−0.00 (0.0003) p = 0.3570
Volume first week	0.06 (0.01) **p = 0.0009**	0.03 (0.01) **p = 0.0031**	0.00 (0.002) p = 0.9384

Estimates (SE); p-value are from GEE models adjusted for treatment group, sex, GA and site. All available data from the MRI mITT sample (n = 207) was used for these analyses. Any transfusion compares patients with at least 1 transfusion to those with none.

## Discussion

### Epo and perinatal variables

Anemia is a physiological stimulant of Epo production, so as anticipated, patients with lower initial hematocrit levels had higher baseline Epo concentrations. Higher baseline Epo were also associated with lower gestational age in all patients, a relationship that persisted after adjusting for hematocrit. At one-week, higher Epo concentrations in the placebo group were associated with lower birth weight. Higher endogenous Epo concentrations appear to be associated with the smallest, most immature of infants. A lack of adequate Epo response has been cited as a precursor for anemia of prematurity [7], however our findings suggest that even infants at 24 weeks have a robust Epo response at birth. This response is not secondary to anemia alone; it appears to be either intrinsic or possibly related to hypoxic stress associated with premature birth.

Higher baseline Epo correlated with lower Apgar scores at 1-minute in all patients. In the placebo group, higher 2-week Epo AUC correlated with lower 1- and 5-minute Apgar scores. These findings suggest that hypoxic stress may have been a triggering factor for the higher endogenous Epo concentrations.

Consistent with this idea is our finding that delayed cord clamping was associated with lower baseline Epo concentrations, even after adjusting for hematocrit. This may be related to the improved perfusion noted with delayed cord clamping [22,23], which would lead to less tissue hypoxia and less hypoxia-induced Epo response. This is the first report to our knowledge to discuss such an association.

### Epo and retinopathy of prematurity

In our analysis, neither endogenous Epo at birth nor its trajectory from birth to 14 days in the placebo group affected ROP outcomes, after controlling for anemia. This contradicts our earlier findings in a small prospective cohort study (n = 27) that showed that higher Epo levels at birth and in the first week of life were associated with more severe ROP [13] and with findings of the ELGAN study of extremely preterm newborns (n = 867), in which an increased risk of severe ROP was observed for the highest 14-day EPO quartile [2]. However, our result is consistent with other studies that showed no correlation of early Epo with ROP after adjusting for GA and anemia [20,24].

We also observed no association between treatment with rHuEpo and ROP, and interaction tests of our hypothesis that treating infants who had high endogenous Epo with rHuEpo increases the risk for ROP was also negative. Our results are consistent with the Cochrane review of early erythropoiesis-stimulating agents (including N = 1283 from 8 studies) [25] and previously published results for the PENUT study [14,26]. However, the Cochrane review comparing early to late rHuEpo treatment (N = 191 from 2 studies) observed an increased risk of ROP in the early treatment group (RR 1.45, 95% CI 1.08–1.95) [27].

### Transfusions and retinopathy of prematurity

This post-hoc analysis confirmed previously established relationships between pRBC transfusions and ROP; however, it was the first to identify a sex-specific correlation, identifying higher rates of ROP among males who received higher volumes of transfusions in the first week after birth but not among females. This sex-specific correlation has clinical implications with the potential to alter transfusion practices for the prevention of ROP but requires further confirmation and investigation into mechanisms of action. We tested the hypothesis that infants with higher endogenous Epo who then received transfusions would have a higher risk for ROP by performing an interaction test between endogenous Epo with transfusions and ROP outcomes. However, this interaction test was non-significant for all subjects, placebo subjects, and rHuEpo-treated subjects. This suggests that the association between transfusions and ROP outcomes is likely independent of endogenous Epo level at birth.

### Epo and brain injury on MRI

Magnetic resonance imaging (MRI) near term gestation is recognized as a useful tool for assessing regional brain injury and is shown to correlate with later neurodevelopment [19,28]*.* Mayock et al demonstrated in the same population (PENUT) of the present study, that evidence of brain injury on MRI at near term PMA correlates with adverse 2-year neurodevelopmental outcomes as assessed by BSID-III [19]. Identifying children at higher risk for neurodevelopmental impairments by MRI prior to NICU discharge allows an opportunity for early intervention [29] and possible improved cognitive and motor functions [30].

In this post hoc analysis, associations between Epo concentrations and MRI results differed between treatment and placebo groups and within white and grey matter.

Consistent with our hypothesis that higher early endogenous Epo (a biomarker for hypoxia) would be associated with increased brain injury, our analysis shows that higher Epo concentrations over the first two weeks of life in the placebo group correlated with increased white matter injury (AUC_[0-14d]_ r = 0.28, p = 0.02). In contrast, higher Epo concentrations at 14 days in the treatment group (reflecting mostly exogenous rHuEpo and to a lesser extent endogenous Epo) correlated with less white matter injury. It is important to note that at 14 days the treatment group had about 8 times higher Epo concentrations than the placebo group (based on the natural log transformed value in Table 4), which would be expected to protect developing white matter [31,32]. Epo exerts its neuroprotective effects by anti-excitotoxic, antioxidant, anti-inflammatory and anti-apoptotic effects [31], and recombinant Epo has been shown to prevent or mitigate white matter injury in animal models, with a particularly beneficial effect on pre-oligodendrocytes [33]. Another contributing factor is that rHuEpo treatment decreases transfusions, which in our analysis were associated with white matter injury. Our results complement findings by Leuchter et al [34], who showed decreased white matter injury on MRI at term equivalent age among preterm infants that were given early high dose rHuEpo.

An unexpected finding was the association of higher baseline Epo concentrations with less grey matter injury on MRI at 36 weeks PMA in the placebo group but increased grey matter injury in the treatment group. The reason for this observation is unclear. In preterm neonates, white matter is generally considered the primary and initial site of injury in response to hypoxia-ischemia, due to the highly sensitive pre-oligodendrocytes (which particularly benefit from rHuEpo [33]) that are responsible for producing myelin; gray matter injury is often a secondary consequence that arises after connected axons are disrupted [32,33,35,36]. Epo and its receptor are expressed in the human brain very early in fetal development [37], and Epo is robustly upregulated in response to fetal hypoxia [1]. Given the significant differences between white and gray matter in timing of development and susceptibility to hypoxic-ischemic injury, and in their responsiveness to rHuEpo, we speculate that they have dissimilar responses to high systemic Epo (from rHuEpo administration) and local feedback loops driving or suppressing Epo production and receptor activation in the brain. Since data are available only for serum Epo concentrations, local cerebral concentrations are unknown. The relative contribution of recombinant vs. endogenous Epo, potential differences in negative feedback on local endogenous production, and differences in receptor activation cannot be assessed. We report an interesting initial observation, but further investigation will be needed to elucidate the underlying mechanism.

Understanding the associations between Epo concentrations and brain injury via MRI may shed light on the delicate balance of how different areas of the developing brain are affected by hypoxia/stress and the protective effects of endogenous Epo and exogenous rHuEpo. To our knowledge, this study is the first to point out such associations.

### Transfusions and brain injury on MRI

Very few studies to date have used MRI to study the impact of pRBC transfusions on brain injury in premature infants, particularly with brain MRI near term PMA. The number of pRBC transfusions during NICU stay correlated with increased white matter injury in our prior cohort study of preterm infants (n = 27), and transfused females but not males had increased total brain injury [13]. Another study (n = 29) focused primarily on sex-specific associations between early brain structure and function with pre-transfusion Hb after pRBC transfusion [38]. In that study, pre-transfusion Hb positively correlated with increased white matter volume in males but not females and negatively correlated with 12-month BSID-III gross motor scores and pooled mean score in females but not males [38]. A third study evaluated MRI of children aged 12–13 years (N = 44) that had been randomized as premature infants to liberal or restrictive transfusion thresholds [39]. Substantially smaller brain volumes were observed in the liberal group compared with controls, while volumes in the restricted group were similar to control; cerebral white matter was substantially reduced in both groups but more so in the liberal group, and females had the most significant abnormalities [39,40].

Our results add significantly to this small body of literature. The number of transfusions received and the overall pRBC volumes received positively correlated with increased total brain injury score and with white matter injury score on MRI obtained near term PMA. However, the association between transfusions and MRI score did not differ by treatment group or by sex in this analysis of a much larger sample size. Additionally, our data, to our knowledge, is the first to show that the total volume of pRBC transfusions received during the first week of life correlated with increased brain injury and white matter injury on MRI. This observed correlation of transfusions with increased brain injury presages the association of transfusions with adverse 2-year neurodevelopmental outcomes that has previously been reported for the same population (PENUT) by Vu et al [9]. All components of BSID-III scores at 2 years progressively decreased as a function of number of transfusions, cumulative volume of transfusions, and number of donor exposures [9].

Transfusion volumes in the first week of life were similar between rHuEpo treatment and placebo groups; this is not unexpected, as previous studies [41] have shown that in a lamb model of rHuEpo administration, it can take 10 days after a massive dose of rHuEpo to see significant impact on hemoglobin. Additionally, the positive rHuEpo effect on hemoglobin pales in comparison to the pressure on the red cell mass due to phlebotomy in the first week of life.

### Endogenous Epo and other outcomes

We observed an association between higher baseline Epo concentrations and an increased risk of death in all patients. Additionally, higher baseline Epo concentrations were associated with increased incidence of IVH and of grade III and IV hemorrhage. There have been inconsistent reports regarding this association. Consistent with our results, several have reported a positive association, including Khosravi et al (N = 50 at 27–37 weeks GA exposed to chorioamnionitis) [42], Efstathiou et al (N = 47 at 28–33 weeks GA) [43], and Bhandari et al (N = 116 at 23–34 weeks GA) [44], but others reported no associations, e.g., Najib et al (N = 140 at 26–34 weeks GA) [45]. Our much larger sample size supports a positive association.

No associations were observed between early endogenous Epo concentrations and BSID-III composite scores at two-years CA, as previously reported for this population by Woods et al [6]. Our failure to observe a significant association in this population may be due to timing of neurodevelopmental assessment at 2 years, since in a 10-year assessment of neurocognition in the ELGAN study (N = 873), elevated early Epo concentrations were associated with IQ values that were >2 SD below the expected mean, with OR (95% CI) of 2.2 (1.3, 3.5) [5].

### Limitations

This post hoc retrospective analysis is limited by the fact that the original randomized trial supplying the raw data was not designed or powered to answer these questions. Furthermore, as in any observational analysis, confounding variables may complicate interpretation of data. Since this analysis was exploratory in nature, no statistical adjustments were made for multiple comparisons. Strengths include the large number of enrolled patients, thoroughness of the data, and retention rates.

## Conclusions

Endogenous early Epo concentrations correlate with white matter injury, IVH, and death, but do not correlate with ROP. The association of Epo with white and grey matter injury, as assessed by near term MRI, differs between placebo and rHuEpo treatment groups, an exploratory finding that may inform additional studies. Transfusions were associated with increased risk and severity of ROP and brain injury on MRI.

## Supporting information

S1 Appendix Baseline maternal, pregnancy/delivery and infant characteristics of patients enrolled in the PENUT trial. (PDF)

S2 Appendix Association of pregnancy characteristics with Epo concentrations over time (GEE models). (PDF)

S3 Appendix Spearman’s correlation of Epo with perinatal variables and outcomes, at baseline in all patients and in the placebo group over time (WITHOUT adjustment for GA). (PDF)

S4 Appendix Spearman’s correlation of Epo vs perinatal variables and outcomes in the treatment group (WITHOUT adjustment for GA). (PDF)

S5 Appendix Interactions of Epo with ROP. (PDF)

S6 Appendix Interactions of Epo with brain injury outcomes by MRI (GEE models). (PDF)

S7 Appendix Interaction of Baseline Epo and transfusions with ROP outcomes. (PDF)
