# Supplementary material for: Erythropoietin, transfusions, and outcomes of retinopathy of prematurity and brain injury in extremely preterm infants: A post hoc analysis of the Preterm Erythropoietin Neuroprotection Trial (PENUT)
Source: PLoS One. 2026 Jun 25;21(6):e0348061. doi: 10.1371/journal.pone.0348061 (PMC13298946; doi:10.1371/journal.pone.0348061)
Supplement: S1 Appendix — (PDF) [file pone.0348061.s001.pdf]

S1 Appendix. Baseline maternal, pregnancy/delivery and infant characteristics of patients enrolled in the PENUT trial (GEE models), adapted from Juul et al, N Engl J Med. 2020;382: 233–243.

Characteristic	Erythropoietin (N = 476)	Placebo (N = 460)
**Maternal characteristics**		
Age – yr	29.1±6.2	28.8±6.2
Hispanic ethnic group - no. (%)†	104 (22)	96 (21)
Race - no. (%)†		
White	309 (65)	302 (66)
Black	131 (28)	109 (24)
Unknown or not reported	36 (8)	49 (11)
Education - no (%)		
High school or less	148 (31)	159 (35)
Some college	153 (32)	132 (29)
College degree or greater	113 (24)	119 (26)
Unknown or not reported	62 (13)	50 (11)
**Pregnancy characteristics**		
Maternal indications for delivery - no. (%)‡	76 (16)	76 (17)
Risk of infection - no. (%)§<br>Pregnancy-induced hypertension - no. (%)	349 (73)<br>39 (8)	340 (74)<br>32 (7)
Prenatal glucocorticoid use - no. (%)	430 (90)	412 (90)
Prenatal magnesium sulfate use - no. (%)	374 (79)	375 (82)
Delivery complications - no. (%)¶	79 (17)	70 (15)
Cesarean delivery - no. (%)	337 (71)	314 (68)
Delayed cord clamping - no./total no. (%)	171/346 (49)	147/334 (44)
Pregnancy with multiple fetuses - no. (%)	125 (26)	118 (26)
**Infant characteristics**		
Female sex - no. (%)	219 (46)	229 (50)
Gestational age at birth - no. (%)		
24 wk	113 (24)	119 (26)
25 wk	121 (25)	124 (27)
26 wk	103 (22)	118 (26)
27 wk	139 (29)	99 (22)
Mean gestational age at birth - wk	26.0±1.2	25.8±1.1
Weight - g	806.4±194.6	792.9±182.2
Weight <10th percentile for gestational age - no. (%)	69 (14)	78 (17)
Head circumference <10th percentile - no. (%)	80 (17)	81 (18)
Apgar score at 5 min	6.1±2.2	6.2±2.1
Apgar score <5 at 5 min - no. (%)	104 (22)	85 (18)
Intracranial hemorrhage before first infusion - no. (%)	100 (21)	94 (20)
Median age at first infusion (interquartile range) - hr	21.1 (15.3–23.5)	20.0 (14.8–23.3)

Plus–minus values are means ±SD. Percentages may not total 100 because of rounding. Abbreviations: No. – Number. †Hispanic ethnic group and race were reported by the mother.‡Maternal indications for delivery were defined as eclampsia, preeclampsia, or seizures. §Risk of infection was defined as pyrexia, chorioamnionitis, prolonged rupture of membranes, administration of antibiotic agents, or preterm labor. ¶Defined as the presence of one or more of the following complications during delivery: prolapsed cord, true knot, tear or rupture of cord, placental abruption, twin-twin transfusion, fetal or maternal bleeding, ruptured uterus, or traumatic instrument delivery.
