# Supplementary material for: Erythropoietin, transfusions, and outcomes of retinopathy of prematurity and brain injury in extremely preterm infants: A post hoc analysis of the Preterm Erythropoietin Neuroprotection Trial (PENUT)
Source: PLoS One. 2026 Jun 25;21(6):e0348061. doi: 10.1371/journal.pone.0348061 (PMC13298946; doi:10.1371/journal.pone.0348061)
Supplement: S2 Appendix — (PDF) [file pone.0348061.s002.pdf]

S2 Appendix. Association of pregnancy characteristics with Epo concentrations over time (GEE models)

	Baseline Epo (Both groups)	Day 7 Epo (Placebo group)	Day 9 Epo (Placebo group)	Day 14 Epo (Placebo group)	Epo $AUC_{[0-14d]}$ (Placebo group)
Maternal age	-0.01 (0.01)<br>p=0.5982	0.00 (0.01)<br>p=0.7265	-0.02 (0.02)<br>p=0.2218	0.01 (0.01)<br>p=0.6795	-0.00 (0.01)<br>p=0.9123
Maternal race – white	0.15 (0.13)<br>p=0.2656	-0.00 (0.15)<br>p=0.9914	-0.17 (0.25)<br>p=0.5132	-0.14 (0.19)<br>p=0.4788	-0.01 (0.16)<br>p=0.9701
Pregnancy-Induced Hypertension	0.00 (0.18)<br>p=0.9893	0.40 (0.20)<br>p=0.0614	0.41 (0.41)<br>p=0.3239	0.32 (0.24)<br>p=0.1955	0.41 (0.20)<br>p=0.0502
Cesarean delivery	0.06 (0.14)<br>p=0.6646	0.19 (0.13)<br>p=0.1438	-0.09 (0.19)<br>p=0.6318	0.10 (0.17)<br>p=0.5659	0.23 (0.14)<br>p=0.1119
Pregnancy with multiple fetuses	0.29 (0.15)<br>p=0.0504	0.09 (0.15)<br>p=0.5520	0.10 (0.18)<br>p=0.5846	-0.39 (0.25)<br>p=0.1270	0.19 (0.15)<br>p=0.2131

Estimates and p-values are from GEE models using the natural logarithm of Epo concentration at each time point. These models account for potential correlation within siblings and are adjusted for gestational age at birth and site. For the analysis of baseline Epo using the combined groups, treatment group was also included as a fixed effect.
