# Supplementary material for: Erythropoietin, transfusions, and outcomes of retinopathy of prematurity and brain injury in extremely preterm infants: A post hoc analysis of the Preterm Erythropoietin Neuroprotection Trial (PENUT)
Source: PLoS One. 2026 Jun 25;21(6):e0348061. doi: 10.1371/journal.pone.0348061 (PMC13298946; doi:10.1371/journal.pone.0348061)
Supplement: S3 Appendix — (PDF) [file pone.0348061.s003.pdf]

S3 Appendix. Spearman's correlation of Epo with perinatal variables and outcomes, at baseline in all patients and in the placebo group over time (WITHOUT adjustment for GA)

	Baseline Epo (Both groups)	Baseline Epo (Placebo group)	Day 7 Epo (Placebo group)	Day 9 Epo (Placebo group)	Day 14 Epo (Placebo group)	Epo $AUC_{[0-14d]}$ (Placebo group)
BW	r=-0.01 p=0.7127 n=777	r=-0.01 p=0.7800 n=386	r=-0.06 p=0.2791 n=305	r=-0.12 p=0.1863 n=133	r=0.04 p=0.6941 n=122	r=-0.07 p=0.2469 n=310
GA	**r=-0.14 p<0.0001** n=777	**r=-0.11 p=0.0311** n=386	r=0.10 p=0.0745 n=305	r=-0.05 p=0.5924 n=133	r=0.06 p=0.4948 n=122	r=-0.05 p=0.3489 n=310
First HCT (within first week)	**r=-0.12 p=.0011** n=775	**r=-0.23 p<.0001** n=385	r=0.03 p=0.5506 n=304	r=-0.09 p=0.3079 n=132	r=0.11 p=0.2420 n=121	r=-0.10 p=0.0870 n=309
Apgar 1	**r=-0.13 p=0.0004** n=774	**r=-0.20 p<.0001** n=385	r=0.06 p=0.2954 n=305	r=-0.10 p=0.2500 n=133	r=-0.12 p=0.1894 n=122	**r=-0.14 p=0.0111** n=310
Apgar 5	**r=-0.09 p=0.0089** n=775	**r=-0.10 p=0.0407** n=386	r=0.03 p=0.6226 n=305	r=-0.10 p=0.2600 n=133	r=-0.14 p=0.1149 n=122	**r=-0.12 p=0.0279** n=310
ROP Stage (max of both eyes)	r=0.07 p=0.0500 n=700	r=0.03 p=0.5509 n=353	r=-0.04 p=0.4752 n=295	r=0.10 p=0.2502 n=130	r=-0.10 p=0.2688 n=120	r=0.05 p=0.4199 n=300
MRI Total Injury Score	r=0.05 p=0.5292 n=168	r=-0.04 p=0.7381 n=87	r=0.14 p=0.2439 n=68	r=0.18 p=0.2139 n=50	r=0.10 p=0.5579 n=40	r=0.08 p=0.4914 n=72
MRI White Matter Score	r=0.03 p=0.6768 n=168	r=0.03 p=0.7897 n=87	**r=0.26 p=0.0354** n=68	r=0.12 p=0.3934 n=50	r=0.06 p=0.6976 n=40	**r=0.29 p=0.0151** n=72
MRI Grey Matter Score	r=0.01 p=0.9217 n=168	**r=-0.27 p=0.0101** n=87	r=-0.19 p=0.1186 n=68	r=0.11 p=0.4371 n=50	r=-0.06 p=0.7148 n=40	r=-0.18 p=0.1284 n=72

Spearman partial correlation coefficient estimate and p-value are presented for association of ln(Epo) at birth for all patients and in the placebo group at each time point with perinatal variables and ROP and MRI outcomes, with no adjustment for gestational age (GA). Analysis subset includes only subjects with Epo values at the respective time points (and at least 2 values for AUC). Abbreviations: AUC: area under the curve; ROP: retinopathy of prematurity.
