# Supplementary material for: Erythropoietin, transfusions, and outcomes of retinopathy of prematurity and brain injury in extremely preterm infants: A post hoc analysis of the Preterm Erythropoietin Neuroprotection Trial (PENUT)
Source: PLoS One. 2026 Jun 25;21(6):e0348061. doi: 10.1371/journal.pone.0348061 (PMC13298946; doi:10.1371/journal.pone.0348061)
Supplement: S4 Appendix — (PDF) [file pone.0348061.s004.pdf]

S4 Appendix. Spearman's correlation of Epo vs perinatal variables and outcomes in the treatment group (WITHOUT adjustment for GA)

	Baseline Epo (Treatment group)	Day 7 Epo (Treatment group)	Day 9 Epo (Treatment group)	Day 14 Epo (Treatment group)	Epo $AUC_{[0-14d]}$ (Treatment group)
BW	r=-0.01 p=0.7860 n=391	**r=0.16 p=0.0031** n=358	r=0.03 p=0.7329 n=179	r=-0.02 p=0.7462 n=193	**r=0.17 p=0.0013** n=357
GA	**r=-0.17 p=0.0006** n=391	r=0.09 p=0.1082 n=358	r=-0.13 p=0.0753 n=179	r=-0.04 p=0.5590 n=193	r=0.08 p=0.1193 n=357
First HCT (within first week)	r= -0.00 p=0.9961 n=390	**r= 0.13 p=0.0142** n=357	r= 0.09 p=0.2558 n=178	r= 0.12 p=0.0965 n=192	**r= 0.14 p=0.0095** n=356
Apgar 1	r=-0.06 p=0.2030 n=389	r=0.00 p=0.9899 n=356	r=0.06 p=0.4464 n=178	r=-0.09 p=0.2155 n=191	r=0.02 p=0.7036 n=355
Apgar 5	r=-0.08 p=0.0975 n=389	r=0.04 p=0.4137 n=356	r=0.13 p=0.0933 n=178	r=-0.04 p=0.5474 n=191	r=0.05 p=0.3057 n=355
ROP Stage (max of both eyes)	**r=0.12 p=0.0294** n=347	r=-0.08 p=0.1267 n=331	r=0.01 p=0.8943 n=170	r=0.08 p=0.2859 n=187	r=-0.05 p=0.3735 n=331
MRI Total Injury Score	r=0.14 p=0.2073 n=81	r=-0.11 p=0.3215 n=77	r=-0.16 p=0.2314 n=60	r=-0.21 p=0.0860 n=67	r=-0.09 p=0.4025 n=80
MRI White Matter Score	r=0.04 p=0.6966 n=81	r=-0.03 p=0.8240 n=77	r=-0.09 p=0.5115 n=60	**r=-0.34 p=0.0053** n=67	r=-0.02 p=0.8536 n=80
MRI Grey Matter Score	**r= 0.23 p=0.0382** n=79	r=-0.15 p=0.2062 n=75	r=-0.21 p=0.1036 n=59	r=0.23 p=0.0633 n=66	r=-0.08 p=0.4927 n=78

Spearman partial correlation coefficient estimate and p-value are presented for association of ln(Epo) in the rHuEpo-treatment group at birth and at each time point with perinatal variables and ROP and MRI outcomes, with no adjustment for gestational age (GA). Analysis subset includes only subjects with Epo values at the respective time points (and at least 2 values for AUC). Abbreviations: AUC: area under the curve; ROP: retinopathy of prematurity.
