# Supplementary material for: Erythropoietin, transfusions, and outcomes of retinopathy of prematurity and brain injury in extremely preterm infants: A post hoc analysis of the Preterm Erythropoietin Neuroprotection Trial (PENUT)
Source: PLoS One. 2026 Jun 25;21(6):e0348061. doi: 10.1371/journal.pone.0348061 (PMC13298946; doi:10.1371/journal.pone.0348061)
Supplement: S5 Appendix — (PDF) [file pone.0348061.s005.pdf]

S5 Appendix: Interactions of Epo with ROP

Table S5a: Interaction of Baseline Epo and treatment with ROP outcomes

	Severe ROP	ROP
Treatment by Baseline Epo	P=0.5837	P=0.5096

P-values are from GEE models adjusted for sex, GA and site. All available data from the mITT sample was used for these analyses.

Table S5b: Epo Trajectory and ROP outcomes in the Placebo Group

	Severe ROP	ROP
Day 14 Epo by Baseline Epo interaction (Placebo only)	P=0.7221	P=0.6105
Day 14 Epo by Baseline Epo interaction (Placebo only) and adjusted for hematocrit	P=0.7082	P=0.5858

P-values are from GEE models adjusted for sex and GA. All available data from the mITT sample was used for these analyses.

Table S5c: Comparing ln(Epo) and ROP binary outcomes in both groups at baseline and placebo group at each time point

	Baseline Epo (Both groups)	Baseline Epo (Placebo group)	Day 7 Epo (Placebo group)	Day 9 Epo (Placebo group)	Day 14 Epo (Placebo group)	Epo $AUC_{[0-14d]}$ (Placebo group)
Any ROP (> 0 in either eye)	p=0.2372	p=0.9189	p=0.7285	p=0.1421	p=0.2394	p=0.8478
Severe ROP (3 or 4 or plus)	p=0.0557	p=0.4139	p=0.5552	p=0.3433	p=0.6959	p=0.2106

P-values in table are from two group t-tests comparing mean ln(Epo) between absence and presence of ROP (or Severe ROP).

Table S5d: Comparing ln(Epo) and ROP binary outcomes in treatment group at different time periods

	Baseline Epo (Treatment group)	Day 7 Epo (Treatment group)	Day 9 Epo (Treatment group)	Day 14 Epo (Treatment group)	Epo $AUC_{[0-14d]}$ (Treatment group)
ROP (> 0 in either eye)	p=0.1248	p=0.2547	p=0.5865	p=0.7204	p=0.7323
Severe ROP (3 or 4 or plus)	p=0.0589	p=0.8566	p=0.2040	p=0.0821	p=0.9741

P-values in table are from two group t-tests comparing mean of ln(Epo) between absence and presence of ROP (or Severe ROP).
