# Supplementary material for: Erythropoietin, transfusions, and outcomes of retinopathy of prematurity and brain injury in extremely preterm infants: A post hoc analysis of the Preterm Erythropoietin Neuroprotection Trial (PENUT)
Source: PLoS One. 2026 Jun 25;21(6):e0348061. doi: 10.1371/journal.pone.0348061 (PMC13298946; doi:10.1371/journal.pone.0348061)
Supplement: S6 Appendix — (PDF) [file pone.0348061.s006.pdf]

S6 Appendix. Interactions of Epo with brain injury outcomes by MRI (GEE models)

Table S6a: Associations between ln(Epo) and Brain Injury on MRI in the Placebo group (GEE).

	Baseline Epo (both groups combined)	Baseline Epo Placebo	Day 7 Epo Placebo	Day 9 Epo Placebo	Day 14 Epo Placebo	$AUC_{Epo[0-14d]}$ Placebo
MRI Total Injury Score	0.14 (0.10) p=0.1708	0.08 (0.27) p=0.7628	0.20 (0.35) p=0.5072	0.34 (0.21) p=0.1887	0.01 (0.54) p=0.9926	0.02 (0.31) p=0.9498
MRI White Matter Injury Score	0.10 (0.06) p=0.0988	0.23 (0.15) p=0.1355	0.28 (0.23) p=0.0844	0.16 (0.13) p=0.2395	0.02 (0.25) p=0.9297	0.32 (0.20) p=0.0687
MRI Grey Matter Injury Score	0.00 (0.01) p=0.9822	**-0.07 (0.02) p=0.0272**	-0.02 (0.02) p=0.0580	0.02 (0.02) p=0.3822	0.01 (0.04) p=0.8328	-0.03 (0.02) p=0.0931

Estimates, SE and p-values are from GEE models for associations between MRI scores and ln(Epo) in both groups combined and in the placebo group at each time point and AUC. These models account for potential correlation within siblings and are adjusted for gestational age at birth and site. For the analysis of baseline Epo using the combined groups, treatment group was also included as a fixed effect.

Table S6b: Associations between ln(Epo) and Brain Injury on MRI in the Treatment group (GEE).

	Baseline Epo (Treatment group)	Day 7 Epo (Treatment group)	Day 9 Epo (Treatment group)	Day 14 Epo (Treatment group)	$AUC_{Epo[0-14d]}$ (Treatment group)
MRI Total Injury Score	0.16 (0.09) p=0.0904	-0.28 (0.11) p=0.0819	-0.13 (0.17) p=0.4318	-0.45 (0.28) p=0.1241	0.17 (0.14) p=0.2700
MRI White Matter Score	0.04 (0.05) p=0.4759	-0.11 (0.07) p=0.2708	-0.02 (0.11) p=0.8939	-0.30 (0.14) p=0.0517	0.09 (0.08) p=0.2271
MRI Grey Matter Score	0.02 (0.01) p=0.0676	-0.03 (0.03) p=0.2589	-0.04 (0.02) p=0.0729	0.04 (0.03) p=0.1211	-0.02 (0.02) p=0.3229

Estimates, SE and p-values are from GEE models for associations between MRI scores and ln(Epo) in the rHuEpo group at each time point and AUC. These models account for potential correlation within siblings and are adjusted for gestational age at birth and site.
