# Supplementary material for: Erythropoietin, transfusions, and outcomes of retinopathy of prematurity and brain injury in extremely preterm infants: A post hoc analysis of the Preterm Erythropoietin Neuroprotection Trial (PENUT)
Source: PLoS One. 2026 Jun 25;21(6):e0348061. doi: 10.1371/journal.pone.0348061 (PMC13298946; doi:10.1371/journal.pone.0348061)
Supplement: S7 Appendix — (PDF) [file pone.0348061.s007.pdf]

S7 Appendix: Interaction of Baseline Epo and transfusions with ROP outcomes.

	Severe ROP	Any ROP
Number of Transfusions by Baseline Epo (both groups)	p=0.5168	p=0.8199
Number of Transfusions by Baseline Epo (Placebo only)	p=0.8266	p=0.4321
Number of Transfusions by Baseline Epo (Treatment only)	p=0.4092	p=0.9211

P-values are from GEE models adjusted for treatment group, sex, GA and site. All available data from the MITT sample was used for these analyses. These are non-significant interactions, i.e. the association between transfusions and ROP outcomes did not differ by baseline Epo level.
